# Including People With Lived Experience in Research From Design to Publication: The Next Steps for the IJIC Community

**DOI:** 10.5334/ijic.10240

**Published:** 2025-11-20

**Authors:** K. Viktoria Stein, Robin Miller, Edelweiss Aldasoro, Michelle Nelson, Eskil Degsell

**Affiliations:** 1 VM Partners Integrating Health and Care and Leiden University Medical Centre, NL; 2 University of Birmingham, UK; 3 International Foundation for Integrated Care (IFIC), UK; 4 Bruyère Health Research Institute, CA.; 5 Swedish Brain Tumor Association, SE

**Keywords:** Co-production, research design, lived experience, publication process, journey mapping

In a recent article by Michgelsen et al. (2025) [1], a key insight emerged from a decade of research published in the International Journal of Integrated Care (IJIC): while the ambition to co-produce integrated care with people is widely stated, the reality often reflects a top-down approach of integrated care onto individuals. This discrepancy is not limited to practice and policy—it permeates the research landscape as well. The resulting gap risks producing research that does not respond to the users’ needs and fails to generate the evidence required to create truly person-centred care. In this editorial, we aim to highlight the challenges and opportunities of co-producing research with people with lived experience, drawing on discussions and outcomes from a workshop held at ICIC25 and the longer journey IJIC has been on for the past 3 years.

## IJIC’s journey of exploration and discovery so far

Since 2022, the International Journal of Integrated Care (IJIC) has taken significant steps towards embedding lived and living experience into its editorial and publication processes as it recognised its role in pushing the scientific research community to adopt more co-productive methods of working. Using the extensive network of the International Foundation for Integrated Care and a research project exploring citizen leadership [2], a collaborative working group involving individuals with lived experience was created in 2023 to discuss how to implement the principles of co-design and co-production in IJIC’s processes. Over the course of a year, the working group discussed different options and came up with recommendations, which were presented to the IJIC Editorial board. This led to an open call for Expressions of Interest for lived experience editors and finally the recruitment in November 2024, of five editors with lived experience, marking a pivotal shift towards co-production in integrated care research and improving research quality. In addition, IJIC has begun updating its guidelines to better support inclusive practices, demanding a declaration and explanation of the level of involvement of people with lived experience and asking reviewers to pay more attention to this topic. These developments reflect a broader commitment to redefining how scientific journals engage with diverse voices and increase perspective density. Along this journey, the regular workshops conducted at the ICIC conferences to gather feedback, input and ideas from the integrated care community have significantly shaped our understanding of how to continue to evolve.

## Reframing the Role of Research(ers) in Co-Production

Discussions within the IJIC editorial board highlighted several persistent challenges in advancing the meaningful participation of people with lived experience in research. Chief among these is the lack of consistent terminology around participation, which hampers clarity and shared understanding. Additionally, the absence of robust measurement tools makes it difficult to demonstrate the value of lived experience contributions. Structural barriers—such as insufficient funding for participatory research, limited familiarity with non-traditional methodologies, and a general lack of expertise among researchers and people with lived experience — further impede progress. Addressing these issues requires a systemic commitment to continuous learning and inclusive practices.

The ICIC25 workshop was the third in a series designed to explore how the integrated care community could address these challenges and become more person-centred, integrated and co-productive. Co-designed by IJIC’s Lived Experience Editors and the Co-Editors-in-Chief using the Double Diamond approach [3] from design thinking, the workshop focused on how to strengthen co-production throughout the research process.

The first phase—problem definition— brought to light a range of systemic and cultural barriers that hinder meaningful engagement with people who have lived experience. These challenges include:

- Systemic and structural barriers: Systemic challenges include the lack of time, curiosity and resources allocated for co-production in research projects and grants. This absence not only devalues involvement but also introduces recruitment bias, as not everyone can afford to participate without compensation for their time and expenses. Power imbalances within health, social care and academic systems further constrain engagement, especially in integrated care research, which often unfolds within hierarchical settings structured around professionals’ needs.
- Conceptual and methodological ambiguity: Another persistent issue is the absence of a consistent definition of lived experience. Questions arise about whether professionals can contribute their own lived experience, how generalizable individual experiences are, and what constitutes valid lived experience. Despite growing global interest in public and patient involvement (PPI), researchers and people with lived experience often lack training in effective co-production methods, leading to ethical dilemmas and insufficient safeguards. These gaps frequently result in tokenistic involvement, where individuals are invited to participate in isolated activities without influencing the research direction.
- Practical and relational obstacles: Researchers themselves face additional challenges. Many in academia and scientific publishing do not recognise data generated from lived experience as a valid form of evidence, and the methodologies used to incorporate it are often viewed as inferior to traditional quantitative approaches. Recruitment practices tend to rely on familiar networks, excluding diverse voices due to perceived time and budget constraints. When co-production is not embedded from the outset, roles remain ambiguous, contributions lack impact, and frustration and mistrust ensue. The use of academic jargon further alienates participants, especially when their research literacy is not considered.
- The role of fear: Underlying these barriers is a pervasive fear—fear of saying the wrong thing, of retraumatisation, of ethical missteps. This fear, often unspoken and invisible, can contribute to the culture of risk-aversion. Rather than confronting these challenges openly, fear often leads to avoidance and inaction.

## Moving Towards Solutions

In the second phase of the Double Diamond approach, participants focused on identifying actionable solutions. For IJIC, two immediate steps were proposed: first, to develop training and support for peer reviewers that clarifies what good co-production to increase research quality looks like, including recommended methods and evaluation criteria; and second, to launch a Call for Papers on “Failed Co-Production” to foster learning from missteps and missed opportunities.

Participants also emphasised the need to define lived experience more clearly, referencing Harrington et al. (2020) [4] for guidance on patient engagement in research. Arnstein’s Ladder of Participation (1969) [5] was suggested as a framework to guide researchers and authors in evaluating levels of involvement, and the SMART framework [6] to look more broadly at citizen science. These definitions should be promoted through IJIC’s website and author/reviewer guidelines to ensure consistent terminology.

More broadly, complex research questions and developing, evaluating, and implementing complex interventions as person-centred integrated interventions demand interdisciplinary approaches. Promoting collaborative and interdisciplinary research as a standard in integrated care would enhance both research quality and the evidence base. Journey mapping was highlighted as a particularly useful methodology for integrating lived experience into research design, as it captures visually people’s journey and identifies relevant touchpoints and systemic barriers that traditional methods may miss.

The workshop also underscored the importance of exchanging experiences, offering targeted training, and fostering openness about the possibilities, challenges and fears associated with co-production and citizen science. However, caution was advised against training people with lived experience to become researchers, as this risks diluting their unique perspectives. The goal is not seen as turning people into academics, but to adapt research methodologies to include and value people’s expertise authentically. Therefore, research methodologies should be adapted to accommodate these perspectives, ensuring involvement throughout the process and using clear, jargon-free language.

## Next steps for IJIC

Taking on these ideas, the IJIC co-editors-in-chief together with our Lived Experience Editors have started on the quest to include lived experience reviewers throughout our journal processes. Another line of exploration is the creation of a new format for submissions of lived experience researchers and the further diversification of the voices represented in our journal. IJIC extends its gratitude to all workshop participants and our Lived Experience Editors for their invaluable contributions. We are committed to advancing these discussions and will continue to report on progress through the journal and future conferences.
